# Current Status of Antibiograms of *Listeria ivanovii* and *Enterobacter cloacae* Isolated from Ready-To-Eat Foods in Alice, South Africa

**DOI:** 10.3390/ijerph9093101

**Published:** 2012-08-29

**Authors:** Mirriam E. Nyenje, Nicoline F. Tanih, Ezekiel Green, Roland N. Ndip

**Affiliations:** 1 Department of Biochemistry and Microbiology, Faculty of Science and Agriculture, University of Fort Hare, PMB X1314, Alice 5700, South Africa; Email:nyenjem@yahoo.com (M.E.N.); nicofriline@yahoo.com (N.F.T.); egreen@ufh.ac.za (E.G.); 2 Department of Microbiology and Parasitology, Faculty of Science, University of Buea, P.O. Box 63, Buea, Cameroon

**Keywords:** foodborne pathogens, *Listeria ivanovii*, *Enterobacter cloacae*, antibiotic resistance, minimum inhibitory concentration, South Africa

## Abstract

This study assessed the antimicrobial susceptibility of 51 *Listeria ivanovii* and 33 *Enterobacter cloacae* strains isolated from various ready-to-eat foods sold in Alice, South Africa. Isolates were identified using standard microbiological tests and further confirmed using API 20E and API Listeria kits. The disc diffusion technique was used to screen for antimicrobial susceptibility against 15 antimicrobials; minimum inhibitory concentration of five antibiotics was determined by the broth dilution method. All the strains of *E. cloacae* (100%) and 96% of *L. ivanovii* isolates were resistant to at least four or more of the antibiotics; nineteen antibiotypes were obtained based on the antibiotics used in the study. Antibiotype A5: A^R^ PG^R ^VA^R ^E^R ^AP^R ^was predominant in both *L. ivanovii* (23.5%) and *E. cloacae* (57.5%) isolates. Marked susceptibility of *Listeriaivanovii* was observed against chloramphenicol, ciprofloxacin, streptomycin and trimethoprim/sulfamethoxazole (100%) each while *E. cloacae* registered 100% susceptibility to ciprofloxacin only. Various percentages of susceptibility was reported to chloramphenicol and gentamicin (91%) each, nalidixic acid (97%) and streptomycin (94%). The MIC_90 _ranged from 0.004–7.5 µg/mL with *E. cloacae* being the most susceptible organism. The study demonstrated the presence of multi-resistant strains of bacteria in ready-to-eat-foods and speculates that these foods could serve as important vehicles transmitting multi-resistant bacteria to humans.

## 1. Introduction

Antimicrobial resistance is currently the greatest challenge worldwide. It decreases the effectiveness of drugs that decrease morbidity and mortality associated with serious and life-threatening infections and thus, compromising human health [1]. Food contamination with antimicrobial resistant bacteria can be a major threat to public health, as the antibiotic resistance determinants can be transferred to other bacteria of human clinical significance. Since the last decade, the prevalence of antimicrobial resistance among foodborne pathogens has increased [2,3], possibly as a result of selection pressure created by the use of antimicrobials in animal health [4].

Although foodborne diseases represent an important health problem, the international impact of foodborne illness is difficult to estimate as large numbers of illnesses remain underreported [5]. On the other hand, these illnesses are sometimes indistinguishable from other diseases because they share common symptoms. Therefore, it is generally accepted in the scientific community, that the true incidence of foodborne diseases is unknown [6]. However, in the USA, foodborne illnesses are estimated to be responsible for some 76 million cases and 3,000 deaths each year [7], whereas in the United Kingdom, foodborne diseases are estimated at 1.7 million per year [8]. Several devastating outbreaks of foodborne diseases have been reported on the African continent, including acute aflatoxicosis in Kenya, which resulted in 317 cases and 125 deaths [9]. Although cholera is preventable and treatable, Africa continues to be plagued with annual outbreaks. In 2005, a total of 131,943 cases and 2,272 deaths were reported to the World Health Organization with Africa accounting for 58% of the global incidence [10]. In 2008, Zimbabwe, reported 98,424 cases and 4,276 deaths [11].

The most common clinical presentation of foodborne diseases takes the form of gastrointestinal symptoms which are generally mild and self-limiting, hence no antimicrobial therapy is required. However, therapy may be life-saving in patients with underlying illness and those with prolonged febrile course of illness in whom invasive illness is suspected [12]. Antimicrobial resistance in some enteric pathogens, for example *Listeria monocytogenes* has been documented, which has negatively affected the management of serious infections caused by these organisms [13].

*Listeria* species are ubiquitous, Gram-positive bacteria that possess characteristics such as the ability to form biofilms, growth at low temperatures, and tolerance of osmotic stress. These factors inevitably contribute to its persistence in and adaptation to the environment. *L. monocytogenes* and *L. ivanovii* are reported to be the causative agents of listeriosis in both humans and animals, with the latter rarely occurring in humans [3]. However, Guillet *et al.* [14] related the few cases of *L. ivanovii* infection to sporadic occurrence of the organism in nature, as the organism is isolated occasionally from animals or environmental sources including food. Furthermore, this organism has been increasingly isolated in food and different environments in some areas [15,16,17,18]. The bacterium was the most prevalent in wastewater and treated final effluents in the same study area of the present study [16]; similarly it was common in cooked food samples in the area [18] suggesting its prevalence in our environment. Listeriosis has been associated with mortality rates of up to 30% in infants and in patients with underlying diseases [19]. The main route of transmission is through consumption of contaminated food. The bacteria can also be acquired by the foetus from its infected mother via the placenta [3]. Listeriosis in immunocompetent adults develops as gastroenteritis which is normally self-limiting, however in immunocompromised individuals, the infection can manifest as septicaemia or meningoencephalitis whereas perinatal infections can lead to abortion, or sepsis and meningitis in the neonates [20]. 

The first choice of treatment for listeriosis is a *β*-lactam antibiotic (e.g., penicillin or ampicillin), alone or in combination with an aminoglycoside (e.g., gentamicin). The association of trimethoprim with a sulfonamide, such as sulfamethoxazole in co-trimoxazole, is considered to be a second choice therapy [19,21,22]. However, *Listeria* strains resistant to penicillin, ampicillin, erythromycin, streptomycin and tetracycline have been reported which dictates judicious use of these drugs [19,23,24]. 

*E. cloacae* occur in water, sewage, soil, food, and as commensal microflora in the intestinal tracts of humans and animals. They are frequently isolated from clinical and food samples as opportunistic pathogens [25]. These organisms are important nosocomial pathogens responsible for local and systemic infections in humans [26]. Outbreaks of *E. cloacae* have been reported in South Africa [27,28] In 1998, an outbreak in the neonatal intensive care unit of a Gauteng hospital was reported and the investigation revealed that six of the isolates were from environmental sources, three from blood cultures of patients and one from the hands of a staff member [27]. Resistant strains among clinical isolates of these organisms have also been documented in hospitalized patients in Pretoria and Johannesberg [28]. A wide variety of drugs are currently used in the treatment of infections caused by this organism. Such antibiotics include aminoglycosides, trimethoprim-sulfamethoxazole, fluoroquinolones and carbapenems. However, there are reports of an increasing prevalence of antimicrobial-resistant *Enterobacteriaceae*, not only pathogenic but also commensal bacteria, suggestive that the community can act as a reservoir, and food can contribute to the spread of these resistant strains [29]. *E. cloacae* have been reported to be resistant to ampicillin, erythromycin, rifampicin and sulfamethoxazole [30]. Some strains have also been found to produce extended-spectrum beta-lactamase and AmpC, conferring resistance to both third and fourth generation cephalosporins [31]. 

Emergence of antimicrobial resistance bacteria has become a serious problem worldwide. While much of the resistance observed in human medicine is attributed to inappropriate use of antibiotics in humans, there is increasing evidence that antimicrobial use in animals selects for resistant foodborne pathogens that may be transmitted to humans as food contaminants [1,32]. Therefore, the present study was carried out to determine the antimicrobial susceptibility profiles of *L. ivanovii* and *E. cloacae* isolated from ready-to-eat foods from Alice, South Africa, in an effort to assess the risks these foods may pose to the community.

## 2. Results and Discussion

### 2.1. Antimicrobial Patterns

All *L. ivanovii* (51) isolates were resistant (total) to vancomycin, penicillin G and erythromycin, but were sensitive to chloramphenicol, ciprofloxacin, streptomycin and sulfamethoxazole/trimethoprim. Of the 51 isolates, 98% were susceptible to nalidixic acid and gentamicin; while susceptibilities of 82% and 55% were reported to trimethoprim and tetracycline respectively; the rest showed either intermediate or total resistance to these antibiotics. Ciprofloxacin was the most active drug. All *E. cloacae* strains (100%) were susceptible to ciprofloxacin while various degrees of susceptibility to nalidixic acid 32 (97%), streptomycin 31 (94%), gentamicin and chloramphenicol 30 (91%) each, were observed. However, the isolates showed marked resistance (100%) to amoxicillin, penicillin G, vancomycin and erythromycin, while 26 (79%) and 19 (58%) of the isolates were resistant to ampicillin and tetracycline respectively (Table 1). The results of this study corroborate other findings [22,32,33]. However these findings are also contrary to those of Odjadjare *et al*. [16] who reported moderate sensitivity to ciprofloxacin (91%), chloramphenicol (87%) and streptomycin (65%) by strains recovered from waste water effluents in the same study area of the current study. This discrepancy may be due to the different sample sources of the isolates which may impact on their adaptation and response to different chemicals and antimicrobial agents. Previous studies on the antimicrobial susceptibility profiles of *Listeria* species have focused mainly on *L.* *monocytogenes* with a dearth of information in the literature on antibiotic susceptibility profiles for *L. ivanovii*. However, this animal pathogen is equally important to man as it has been isolated from infected humans, indicating pathogenic potential for humans [14]. 

**Table 1:** Antimicrobial susceptibility profile of *L. ivanovii* and *E. cloacae* isolated from ready-to-eat foods.

	Number (%)		
Antibiotic	*L. ivanovii* (n = 51)		
	S	I	R
Chloramphenicol (30 µg)	51 (100)	0 (0)	0 (0)
Kanamycin (30 µg)	21 (39)	19 (37)	11 (22)
Amoxacillin (20 µg)	3 (6)	0 (0)	48 (94)
Penicillin G (10 units)	0 (0)	0 (0)	51 (100)
Vancomycin (30 µg)	0 (0)	0 (0)	51 (100)
Ciprofloxacin (5 µg)	51 (100)	0 (0)	0 (0)
Streptomycin (10 µg)	51 (100)	0 (0)	0 (0)
Trimeth/Sulfamethoxazole (1.25 µg)	51 (100)	0 (0)	0 (0)
Nalidixic acid (30 µg)	50 (98)	1 (2)	0 (0)
Trimethoprim (5 µg)	42 (82)	4 (8)	5 (10)
Erythromycin (15 µg)	0 (0)	0 (0)	51 (100)
Neomycin (30 µg)	20 (39)	24 (47)	7 (14)
Tetracycline (30 µg)	28 (55)	0 (0)	23 (45)
Ampicillin (10 µg)	23 (45)	5 (10)	23 (45)
Gentamicin (10 µg)	50 (98)	1 (2)	0 (0)
**Antibiotic**	***Enterobacter cloacae*** **(n = 33)**		
	S	I	R
Chloramphenicol (30 µg)	30 (91)	2 (6)	1 (3)
Kanamycin (30 µg)	27 (82)	3 (9)	3 (9)
Amoxacillin (20 µg)	0 (0)	0 (0)	33 (100)
Penicillin G (10 units)	0 (0)	0 (0)	33 (100)
Vancomycin (30 µg)	0 (0)	0 (0)	33 (100)
Ciprofloxacin (5 µg)	33 (100)	0 (0)	0 (0)
Streptomycin (10 µg)	31 (94)	2 (6)	0 (0)
Trimeth/Sulfamethoxazole (1.25 µg)	26 (79)	0 (0)	7 (21)
Nalidixic acid (30 µg)	32 (97)	1(3)	0 (0)
Trimethoprim (5 µg)	26 (78)	0 (0)	7 (21)
Erythromycin (15 µg)	0 (0)	0 (0)	33 (100)
Neomycin (30 µg)	15 (45)	13 (39)	5 (15)
Tetracycline (30 µg)	14 (42)	0 (0)	19 (58)
Ampicillin (10 µg)	0 (0)	7 (21)	26 (79)
Gentamicin (10 µg)	30 (91)	1 (3)	2 (6)

S, sensitive; I, intermediate; R, resistant.

Multidrug resistance was a common phenomenon observed in all the 51 (100%) *L. ivanovii* and 33 (100%) *E. cloacae* isolates. Nineteen antibiotic resistance patterns were obtained (Table 2). Antibiotype A5: A^R^ PG^R ^VA^R ^E^R ^AP^R^ (amoxicillin, penicillin G, vancomycin, erythromycin, ampicillin) was predominant in both *L. ivanovii* (23.5%) and *E. cloacae* (57.5%) isolates, followed by A3: A^R^ PG^R ^VA^R ^E^R^ (amoxicillin, penicillin G, vancomycin, erythromycin) which was obtained from seven (21.2%) of *E. cloacae* and 11 (22.5%) of *L. ivanovii*. The least resistant patterns for *E. cloacae* (3%) were demonstrated by A15 and A19 while for *L. ivanovii* (1.9%) A2, A4, A7, A11 and A12 respectively demonstrated the same pattern.

The results indicated alarming multi-resistance frequencies to at least four or more of the test antibiotics. This is of major concern to the public as these foods may act as reservoir of resistant strains that can be transmitted to humans upon ingestion of the contaminated food. Similar patterns of resistance have been reported by other authors [24,30,32]. Srinivasan *et al*. [24] found that all *L. monocytogenes* strains isolated from a dairy farm environment were resistant to ampicillin and rifampicin, and some to tetracycline, penicillin and chloramphenicol. Furthermore, Haryani *et al*. [30] in their study observed that all *E. cloacae* strains studied were resistant to six or more antibiotics. 

The results suggest that incidence of antibiotic resistance in *L. ivanovii* is relatively high. It is of great concern that the range of antibiotics to which resistance has been acquired is wide and the expanding range now includes a number of antibiotics used to treat listeriosis (penicillin, vancomycin, erythromycin and ampicillin). This may have future implications for the effective treatment of listeriosis if these resistant strains could be transferred to humans. Interestingly, the study reported high susceptibility of strains to gentamicin (98%) and trimethoprim/sulfamethoxazole (100%), drugs which are considered as second choice therapy [22]. These findings are in agreement with other reports [22,24,34]. 

**Table 2:** Antibiotypes of *E. cloacae* and *L. ivanovii*.

No	Antibiotype	Number (%)		
		*E. cloacae*	*L. ivanovii*	
A1	PG^R ^VA^R ^E^R ^	0 (0)	3 (5.9)	
A2	PG^R ^VA^R ^E^R ^T^R^	0 (0)	1 (1.9)	
A3	A^R^ PG^R ^VA^R ^E^R^	7 (21.2)	11 (22.5)	
A4	K^R ^A^R^ PG^R ^VA^R ^E^R^	0 (0)	1 (1.9)	
A5	A^R^ PG^R ^VA^R ^E^R ^AP^R^	19 (57.5)	12 (23.5)	
A6	A^R^ PG^R ^VA^R ^E^R ^T^R^	0 (0)	5 (9.8)	
A7	K^R^ A^R^ PG^R ^VA^R ^E^R ^AP^R^	0 (0)	1 (1.9)	
A8	K^R^ A^R^ PG^R ^VA^R ^E^R ^T^R^	0 (0)	3 (5.9)	
A9	A^R^ PG^R ^VA^R ^E^R ^T^R^ AP^R^	0 (0)	5 (9.8)	
A10	A^R^ PG^R ^VA^R ^E^R ^NE^R^ T^R^	0 (0)	2 (3.9)	
A11	A^R^ PG^R ^VA^R ^W^R ^E^R ^T^R ^AP^R^	0 (0)	1 (1.9)	
A12	K^R^ A^R^ PG^R ^VA^R ^E^R ^NE^R ^AP^R^	0 (0)	1 (1.9)	
A13	K^R^ A^R^ PG^R ^VA^R ^E^R ^T^R ^AP^R^	0 (0)	2 (3.9)	
A14	K^R^ A^R^ PG^R ^VA^R ^E^R ^NE^R ^T^ R ^	0 (0)	2 (3.9)	
A15	A^R^ PG^R ^VA^R ^W^R^ E^R ^T^ R ^AP^R^	1 (3)	0 (0)	
A16	A^R^ PG^R ^VA^R ^SXT^R ^W^R^ E^R ^T^ R ^AP^R^	3 (9)	0 (0)	
A17	K^R^ A^R^ PG^R ^VA^R ^W^R^ E^R ^NE^R ^T^ R ^AP^R^	0 (0)	2 (3.9)	
A18	K^R^ A^R^ PG^R ^VA^R ^SXT ^R ^W^R^ E^R ^NE^R ^T^ R ^AP^R^ GM^R^	2 (6)	0 (0)	
A19	C^R ^K^R^ A^R^ PG^R ^VA^R ^SXT ^R ^W^R^ E^R ^NE^R ^T^ R ^AP^R^ GM^R^	1 (3)	0 (0)	

A, amoxicillin; C, chloramphenicol; K, kanamycin; PG, penicillin G; VA, vancomycin; SXT, trimethoprim/sulfamethoxazole; W, trimethoprim; E, erythromycin; NE, neomycin; T, tetracycline; AP, ampicillin; GM, gentamicin.

The increases in antibiotic resistance among *Listeria* spp. are in line with the general worldwide pattern of an increasing prevalence of antibiotic resistance, including multiple antibiotic resistances among many groups of bacteria. Multiple drug resistance in these organisms have been attributed to antimicrobial selective pressure and gene transfer mechanisms between and among *Listeria* species and close relatives of the bacteria such as *Enterococcus*, *Streptococcus* and *Staphylococcus* species [21,34].

The present study observed 100% resistance of *E. cloacae* to amoxicillin, penicillin, ampicillin and erythromycin, while for neomycin and tetracycline it was 45% and 42% respectively. The findings are in agreement with those of Haryani *et al*. [30], who observed 100% resistance to ampicillin and erythromycin; however, their study demonstrated various degrees of resistance to streptomycin (85.71%), ciprofloxacin and tetracycline (42.86%) and trimethoprim (28.57%) contrary to the findings of this study where ciprofloxacin recorded 100% sensitivity. Environmental factors as well as differing prescription practices may try to explain these discrepancies.

The emerging antibiotic resistance in the *Enterobacteriaceae* family is a significant problem that requires vigilance and intensified measures to control the further spread of resistance by these important Gram-negative pathogens. Worthy of note is the fact that most research has involved organisms that directly cause disease, focusing less on important contributions by commensal bacteria. Although these organisms generally do not cause disease in immunocompetent people, they can transfer resistance genes to other bacteria in the environment [31]. The European Community therefore recommended the monitoring of antimicrobial resistance in these organisms; *E. cloacae* is one of the few bacteria where monitoring in healthcare facilities is recommended [35]. 

There are reports on similarities in virulence factors between human and food isolates of *Enterococcus* spp., implying that food commensals may be an overlooked aspect of antibiotic resistance [36]. Several studies have tracked resistant zoonotic species using non-pathogenic *E. coli* as an indicator organism. DeFrancesco *et al*. [37] demonstrated that commensal *E. coli* had higher rates of resistance than pathogenic multi-drug resistant *Salmonella* from the same herds, suggesting that commensal bacteria could be used to survey the prevalence of antibiotic resistant bacteria. On the other hand, food animal reservoirs are an important source of antimicrobial resistance, even though it might be difficult to quantify the exact burden, compared with human use. 

### 2.2. Minimum Inhibitory Concentration (MIC_90_) Determination

Of the five antimicrobials tested, *E. cloacae* isolates were more susceptible to ciprofloxacin with MIC_90_ ranging as low as 0.004–3.75 µg/mL. The MIC_90_ of kanamycin, tetracycline, nalidixic acid and chloramphenicol ranged from 0.029–0.117 µg/mL, 0.029–3.75 µg/mL, 0.029–0.937 µg/mL and 0.058–0.937 µg/mL respectively (Table 3). 

**Table 3:** Minimum inhibitory concentration (MIC_90_) of different antibiotics against *E. cloacae* isolates.

Isolate no.	Cip	T	C	K	NA
1-E	0.009	0.029	0.234	0.029	0.234
2-E	0.019	0.468	0.468	0.058	0.234
4-E	0.009	0.117	0.468	0.117	0.058
5-E	0.625	3.75	0.937	ND	0.234
7-E	0.004	0.117	0.234	0.117	0.117
09-2	0.004	0.117	0.468	0.058	0.029
012-2	0.156	0.468	0.468	0.029	0.234
052-2	0.009	0.234	0.937	0.117	0.234
057-2	0.009	0.117	0.468	0.117	0.058
067-2	0.019	0.468	0.468	0.058	0.117
070-2	0.004	0.117	0.937	0.058	0.234
073-2	0.009	0.234	0.468	0.014	0.058
089-2	0.009	0.117	0.058	0.029	0.468
103-1	0.019	0.234	0.468	0.058	0.117
112-3	0.004	0.058	0.937	0.117	0.117
224-3	0.009	0.117	0.468	0.234	0.117
247-2	0.019	0.117	0.637	0.234	0.468
114-2	0.009	0.117	0.058	0.117	0.468
119-2	0.019	0.117	0.468	0.058	0.029
164-1	0.004	0.029	0.468	0.058	0.468
171-1	0.625	ND	0.468	ND	0.937
172-1	0.625	ND	0.937	ND	0.234
177-1	0.019	0.937	0.468	0.058	0.117
194-3	0.009	0.117	0.234	0.029	0.234
198-2	0.004	0.468	0.468	0.058	0.234
205-3	0.004	0.117	0.937	0.117	0.029
208-2	0.156	0.468	0.468	0.117	0.234
217-3	0.312	ND	0.234	0.029	0.468
220-2	0.009	0.468	0.234	0.029	0.234
221-2	0.004	0.234	0.468	0.117	0.029
225-3	0.009	0.234	0.937	0.117	0.117
235-2	0.312	ND	0.468	ND	0.937
251-3	0.156	ND	0.234	0.117	0.029

Cip, ciprofloxacin; T, tetracycline; K, kanamycin; NA, nalidixic acid; C, chloramphenicol; ND, not determined.

On the other hand, the MIC_90_ for *L. ivanovii* isolates, ranged from 0.009–0.625 µg/mL for ciprofloxacin, 0.014–7.5 µg/mL for tetracycline, 0.029–3.75 µg/mL for chloramphenicol, 0.058–7.5 µg/mL for kanamycin and 0.058–7.5 µg/mL for nalidixic acid (Table 4). 

**Table 4:** Minimum inhibitory concentration (MIC_90_) of different antibiotics against *L. ivanovii* isolates.

Isolate no.	Cip	T	C	K	NA
61-2	0.039	1.875	0.937	0.058	3.75
74-1	0.312	0.937	0.468	0.117	7.5
99-2	0.078	0.117	0.234	0.117	0.468
109-1	0.009	0.058	0.468	3.75	0.117
117-2	0.312	0.937	0.468	0.234	1.875
127-2	0.019	0.117	1.875	0.117	0.468
155-2	0.078	1.875	3.75	0.234	0.468
160-1	0.625	0.234	0.468	0.117	1.875
188-2	0.039	0.937	0.234	0.234	3.75
190-2	0.039	0.937	0.937	0.117	0.937
192-2	0.006	0.014	0.937	0.058	0.058
63-1	0.312	0.937	0.234	0.468	1.875
196-1	0.078	0.117	3.75	0.234	0.468
55-1	0.009	1.875	0.468	3.75	1.875
164-1	0.019	0.058	0.937	1.875	1.875
123-1	0.156	0.058	0.468	0.234	0.468
73-1	0.019	1.875	0.937	0.234	0.468
20-2	0.078	1.875	0.937	0.117	3.75
93-2	0.009	0.058	0.029	3.75	0.234
159-1	0.039	0.468	0.937	0.117	0.468
195-1	0.009	1.875	0.468	3.75	1.875
119-2	0.312	0.058	3.75	0.468	3.75
118-2	0.019	3.75	0.234	1.875	1.875
58-2	0.078	1.875	3.75	0.234	0.468
111-1	0.039	0.937	0.468	0.117	0.937
01-2	0.312	0.937	0.468	0.468	1.875
03-2	0.312	0.058	3.75	0.234	3.75
04-2	0.078	0.117	3.75	0.234	0.468
08-2	ND	0.117	0.468	0.468	3.75
16-2	0.156	0.937	0.937	0.234	7.5
17-2	0.156	0.058	0.468	0.234	0.468
20-2	0.039	0.937	0.937	0.234	3.75
27-E	0.039	0.117	0.937	0.937	0.468
32-3	0.009	0.234	3.75	0.234	0.937
37-1	0.625	0.117	0.468	0.468	0.937
198-1	0.312	0.117	3.7	0.234	3.75
38-1	0.156	0.234	0.468	0.117	1.875
39-1	0.039	0.937	0.937	0.234	0.468
41-1	0.019	0.058	0.937	0.937	1.875
42-3	0.009	1.875	0.234	3.75	1.875
43-E	0.625	7.5	0.468	7.5	0.058
44-1	0.156	0.468	0.937	0.234	ND
80-1	0.156	0.468	0.937	0.937	1.875
85-2	0.019	0.234	0.468	0.234	0.468
131-1	0.625	0.117	0.234	0.468	0.937
93-2	0.039	0.117	0.468	0.468	3.75
100-1	0.039	0.468	0.937	0.117	0.468
140-1	0.156	0.058	0.468	0.234	0.468
106-1	0.078	0.937	0.468	0.937	3.75
201-1	0.039	0.117	0.937	0. 468	0.937
189-1	0.078	0.234	3.75	0.234	0.937

Cip, ciprofloxacin; T, tetracycline; K, kanamycin; NA, nalidixic acid; C, chloramphenicol.

*In vitro* antibiotic susceptibility testing of isolates from patients remains an important guide to therapy for clinicians and a useful marker for epidemiological investigations. The adoption of quantitative determinations of antibiotic susceptibility (MIC determination), rather than qualitative methods (*i.e.*, disc diffusion test) may be of importance in identifying small, but potentially clinical significant changes, in antibiotic susceptibility [38]_. _Interestingly, the study reported a MIC_90_ range as low as 0.004–7.5 µg/mL with *E. cloacae* being the most susceptible organism and ciprofloxacin the most active antibiotic. The findings support the disc diffusion results in which all the isolates were markedly sensitive to this drug.

## 3. Experimental Section

### 3.1. Bacterial Strains

A total of 51 *L. ivanovii* and 33 *E. cloacae* strains were isolated from various food samples which comprised of beef and chicken stew, potatoes, rice, vegetables and pies purchased from cafeterias in Alice. The organisms were isolated and identified as previously reported [39] with some modifications. Briefly, 25 g of each sample was weighed and homogenized under aseptic conditions in 225 mL of buffered peptone water. One millilitre of the homogenate was added to a tube containing 9 mL of sterile buffered peptone water and incubated aerobically at 37 °C for 12–24 hours. A loopful of enrichment broth was cultured on Mac Conkey, Eosin-Methylene Blue (EMB) and Colombia blood agar base (Oxoid, Basingstoke, UK) supplemented with 5% horse blood. The plates were incubated aerobically for 24–48 hours at 37 °C.

Isolates were presumptively identified based on colony pigmentation, Gram staining characteristics and oxidase test. Small beta hemolytic colonies on Colombia blood agar, Gram-positive short rods and oxidase negative were presumptive of *Listeria* species while pink colonies on Mac Conkey and metallic sheen colonies on EMB, Gram-negative rods and oxidase negative were presumptive of *Enterobacter cloacae*. Confirmation of isolates used the API 20E and API Listeria kits (Biomerieux, Marcy-l’Etoile, France). The tests were performed as per manufacturer’s instruction for use and data interpretation was performed using the Analytical profile index (API) database (V4.1) with the apiweb^TM^ identification software.

### 3.2. Antibiotic Susceptibility Test

#### 3.2.1. Disc Diffusion Test

Antibiotic susceptibility of the two organisms (*L. ivanovii* and *Enterobacter* *cloacae*) was carried out using disc diffusion method as recommended by the Clinical and Laboratory Standards Institute CLSI [40] on Mueller Hinton agar (Oxoid, Basingstoke,UK) with the following antibiotic discs: ampicillin (25 μg), erythromycin (15 μg), streptomycin (25 μg), chloramphenicol (30 μg), ciprofloxacin (5 μg), amoxicillin (20 μg), nalidixic acid (30 μg), tetracycline (30 μg), trimethoprim (5 μg), vancomycin (10 μg), trimethoprim-sulfamethoxazole (1.25 μg), gentamicin (10 μg), neomycin (30 μg), penicillin G (10 unit), kanamycin (30 μg). All discs were obtained from Mast Diagnostics, Merseyside, United Kingdom.

Three to five well-isolated colonies from nutrient agar (Oxoid, Basingstoke, UK) were transferred into 5 mL normal saline until its turbidity was equivalent to 0.5 McFarland standards. The suspension was streaked uniformly onto Mueller Hinton agar plate with a cotton swab. Antibiotic discs were placed onto the surface of each plate (5 antibiotics/petri dish) using a sterile forceps. After incubation at 37 °C for 24 h, the diameter of growth inhibition zone surrounding each disc was measured and interpreted according to the CLSI [41] recommendation. Currently, there are no interpretative criteria provided by CLSI for *Listeria* spp. Therefore, the present study used the CLSI criteria for *Staphylococci* spp. [32]. *Staphylococcus aureus* NCTC 6571 and *Pseudomonas aeruginosa* ATCC 15442 reference strains were used to test the efficacy of the discs.

#### 3.2.2. Minimum Inhibitory Concentration (MIC_90_) Determination

Determination of MIC_90_ was done using the broth-dilution technique as previously described by Nyenje and Ndip [42]. Ciprofloxacin, tetracycline, chloramphenicol, kanamycin and nalidixic acid powders which demonstrated marked susceptibility in the disc diffusion test and available in our laboratory at the time of the experiments were used. Tetracycline, nalidixic acid and chloramphenicol powders were dissolved in 0.2% DMSO whilst kanamycin and ciprofloxacin were dissolved in sterile distilled water as per manufacturer’s instruction. 

Mueller Hinton broth (100 μL) was dispensed into the wells except the first well of each row which contained 150 μL and the last row of wells which contained distilled water. A 50 μL of the stock antibiotic was dispensed into the first well; a two-fold serial dilution was carried out up to well number 12 from which 100 μL was discarded. Twenty microlitres of standardized bacterial suspension (0.5 McFarland standards) was added to each of the wells except the control wells (control wells contained broth only and distilled water only). An automatic ELISA microplate reader (SynergyMx, Biotek^R^, Thousand Oaks, CA, USA) adjusted to 570 nm was used to measure the absorbance of the plates before and after incubation at 37 °C for 24 hours. The absorbencies were compared to detect an increase or decrease in bacterial growth and the values plotted against concentration. The lowest concentration of the antibiotic resulting in inhibition of 90% bacterial growth was recorded as the MIC_90_. The following reference strains *Staphylococcus aureus* NCTC 6571 and *Pseudomonas aeruginosa* ATCC 15442 were used as control organisms.

## 4. Conclusions

The results demonstrate the presence of multi-resistant strains of bacteria in ready-to-eat foods. Their public health impact remains unknown, but point to the fact that these foods could be important vehicles transmitting multi-resistant bacteria to humans on a daily basis implying that mitigation of drug resistance in foodborne bacteria is likely to be of benefit to human health. 
